# Infertility knowledge and treatment beliefs among African American women in an urban community

**DOI:** 10.1186/s40834-019-0097-x

**Published:** 2019-09-24

**Authors:** Ashley Wiltshire, Lynae M Brayboy, Kiwita Phillips, Roland Matthews, Fengxia Yan, Desiree McCarthy-Keith

**Affiliations:** 1 Morehouse School of Medicine, Department of Obstetrics and Gynecology, 720 Westview Drive, Atlanta, GA 30310 USA; 2 Department of Obstetrics and Gynecology Division of Reproductive Endocrinology and Infertility Women & Infants Hospital, Alpert Medical School of Brown University, 101 Dudley Fl 1, Providence, RI 02905 USA; 3 Morehouse School of Medicine Department of Community Health & Preventive Medicine, 720 Westview Drive, Atlanta, GA 30310 USA

**Keywords:** Infertility, African American, Women, Reproduction

## Abstract

**Background:**

To assess infertility knowledge and treatment beliefs among African American women in an urban community in Atlanta, Georgia.

**Methods:**

This was a cross sectional study at a safety net hospital. A convenience sample of a total of 158 women receiving outpatient obstetrical or gynecologic care from March–April 2017 were recruited. Infertility knowledge and treatment beliefs were assessed using a previously applied and field-tested survey from the International Fertility Decision Making Study.

**Results:**

The mean infertility knowledge score was 38.15% for total subjects. Those with a higher level of education (*p* < 0.0001) and those with paid employment (*p* = 0.01) had a significantly higher level of infertility knowledge. Those who had a history of infertility therapy were significantly more likely to agree with negative treatment beliefs (*p* = 0.01). There was no significant difference in infertility knowledge or treatment beliefs based on age, sexuality, parity or being pregnant at the time of survey completion.

**Conclusions:**

African American women in our urban clinic setting seem to have a limited level of knowledge pertaining to infertility. Further research is needed to understand how differences in knowledge and beliefs translate into infertility care decision-making and future childbearing.

**Electronic supplementary material:**

The online version of this article (10.1186/s40834-019-0097-x) contains supplementary material, which is available to authorized users.

## Introduction

Infertility is a disease of numerous etiologies, which can affect both men and women of every ethnic group and race around the world [1]. Though it is rarely considered a health issue of serious concern, infertility can lead to distress and depression, as well as discrimination and ostracism in certain cultures [2]. An estimated 48.5 million couples worldwide were infertile in 2010 [1]. According to the Centers for Disease Control and Prevention (CDC), approximately 6% of married women of reproductive age are infertile and approximately 12% of reproductive age women, regardless of marital status, have impaired fecundity in the United States (US) [3].

Similar to many other health conditions in the US, infertility is intertwined with multifactorial racial disparities [4]. Studies show that African American (AA) women are disproportionately affected by infertility in terms of prevalence, utilization of treatment, and access to care [4–7]. Even after adjusting for socioeconomic status, risk factors, and pregnancy intent, a US population-based study showed that AA women ages 33–44 years are still two-fold more likely to experience infertility in comparison to Caucasian American (CA) women [4, 6]. This issue has been of even greater concern, as statistical trends have shown that infertility rates were increasing among AA women while simultaneously decreasing among CA women (5).

This racial disparity persists, in regard to pursuit of infertility care and success rates of Assisted Reproductive Technology (ART). It is well documented in the literature that AA women usually have a longer duration of infertility before seeking care and they also pursue medical care for infertility significantly less often than CA women [4–7]. Currently, there are 15 states in the US with varying degrees of ART insurance coverage, as mandated by law [8]. Even in insurance mandated states, AA women seek infertility care less often than CA women [4]. Of the AA women who do seek care, live birth rates after IVF are disproportionately lower [4, 5, 7]. The trend of poor outcomes in AA women is particularly illustrated by the Society for Assisted Reproductive Technology (SART) database [5, 7]. Trend analysis has shown that live births after fresh embryo transfer occurs significantly less often in AA than CA women. The difference was calculated to be as high as 31% between 2000 and 2004 [5]. In an analysis of SART data from 2004 to 2013, AA women had higher rates of SAB and significantly lower rates of live birth after autogolous and third party ART [7].

Numerous studies have confirmed that there is a racial disparity regarding infertility, which prevails through access, pursuit, utilization and success rates of treatment [4–7]. Nevertheless, the answer as to why these disparities exist and persist remains unclear. Just as unclear, is our understanding of what AA women know of the disease. Likewise, when it comes to patient perspectives of infertility and infertility care, there is a dearth of research on this topic with adequate representation of AA women. Therefore, characterizing this subpopulations’ understanding of infertility and infertility management would likely yield a valuable first step in tackling these issues.

Our objectives in this study were to assess infertility knowledge and treatment beliefs specifically among AA women in an urban setting in Atlanta, Georgia. The ultimate goal was to establish a generalized knowledge level and to uncover unique belief patterns within this subpopulation to use as a foundation for educational intervention, as well as a tool to help shape optimum patient care.

## Material and methods

### Study population

**Fig. 1:**
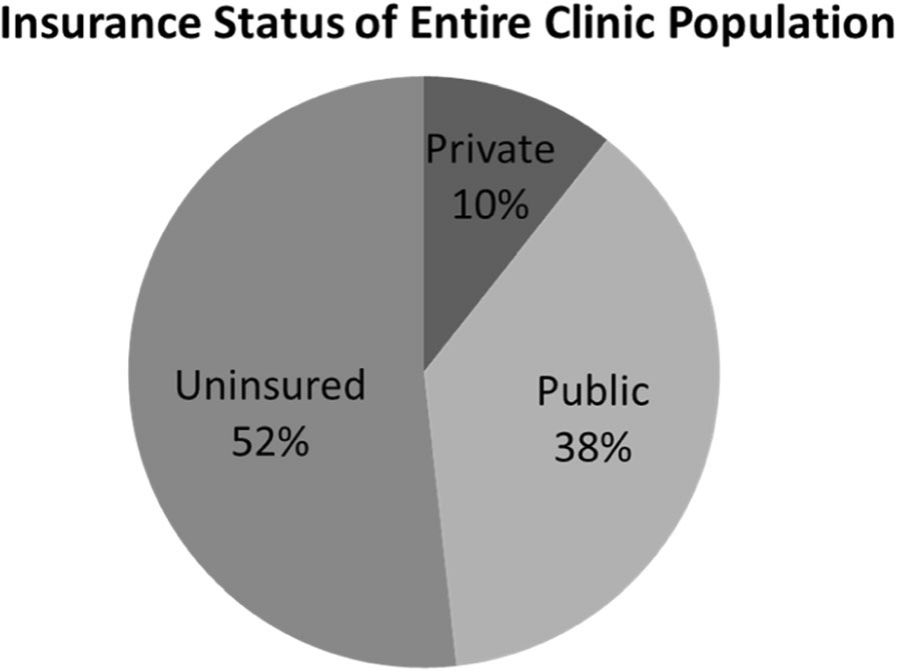
Insurance status of entire clinic population

This study was conducted at a Historically Black College/University (HBCU) affiliated safety-net hospital in an urban community in Atlanta, Georgia. The study participants were recruited from the Obstetrics and Gynecology clinics, staffed by resident physicians, attending physicians, nurse midwives and physician assistants. The insurance status of our patient population at this location is 52% uninsured, 38% public, and 10% private (Fig. 1). From March to April 2017, women presenting for either obstetrical or gynecologic evaluation were recruited to participate in the study. Recruitment was performed by the provider or clinic nurse after the clinic visit was completed to avoid the perception of coercion. Patients were provided with a cover letter describing the study. The cover letter also explained that participation was completely voluntary and would have no impact on their medical care. If the patient decided to participate, a paper copy of the survey was provided for them to complete privately in a separate room before discharge. Inclusion criteria consisted of female gender, age ≥ 18 years, English literacy, and self-identification as Black or African American. Participants were not compensated.

### Survey instrument

The survey instrument consisted of 13 items on infertility knowledge statements answered with True/False/Don’t Know and 6 items of infertility treatment belief statements answered with a Likert scale (Additional file 1). For infertility treatment beliefs, the first two items were considered positive beliefs (ex. “treatment is safe”) and last four items were considered negative beliefs (ex. “treatment is a scary experience”). These 19 questions were taken directly from the published survey administered in the FKBF-IFDM study, with the author’s permission (Fertility Knowledge and Beliefs about Fertility Treatment: Findings from the International Fertility Decision-Making Study). We decided to incorporate these particular infertility knowledge and belief questions because this survey has been previously applied and field tested in 79 different countries, including the US [9]. In addition to those 19 questions, we added an additional 12 questions pertaining to patient demographics and personal obstetrical history. Our final survey was considered to be at the 7th grade reading level per the Flesche-Kincaid scale. It was self-administered and self-paced, requiring approximately 10 min to complete.

### Statistical analyses

The data was coded, logged into a Microsoft Excel and then analyzed using Statistical Analysis Software (SAS 9.4). For infertility knowledge, the patient was given a score of 1 if answered correctly and 0 if the answer was incorrect or if they chose “don’t know”, as done in the original study. The final knowledge score was calculated using summation of the number of correctly answered questions divided by total number of questions and multiplied by 100. The score range was 0–100%. For infertility treatment beliefs, the answer range for both the positive and negative statements were based on a Likert score of 1–5. A selection of “1” signified strongly disagree and “5” signified strongly agree. The mean Likert score was calculated and used for comparisons.

Descriptive statistics was performed to summarize the demographic characteristics and other related measurements. The frequency with percentage was used for categorical variables and the mean with standard deviation was used for numerical variables. Two independent sample t-tests were used to compare the knowledge and belief difference between two level categorical variables (i.e., age, race, education, employment status, pregnant status and parity). An analysis of variance test was used to examine the knowledge and belief differences among three types of sexual orientation. A multiple linear regression analysis was performed to examine the individual effect of different factors by controlling other factors for positive beliefs, negative beliefs and knowledge. All the data analyses were performed using SAS 9.4 and *p* < 0.05 was considered to be statistically significant.

## Results

A total of 158 completed questionnaires were collected meeting our inclusion criteria. The mean age of the subjects was 35 years, with an age range from 18 to 72 years. Fifty-three and a half percent (53.5%) of the total participants were younger than 35 years. Twenty-nine percent (29%) had a higher-level education (university or post-graduate). Fifty-nine percent (59%) had paid employment. Sexual orientation was as follows: heterosexual (90%), homosexual (6%), bisexual (4%). Eleven percent (11%) of subjects reported a history of difficulty conceiving and 3% of total participants required infertility therapy for conception in the past. Forty-seven percent (47%) of participants were pregnant at the time of questionnaire completion. The average age of first pregnancy/delivery was 20 years, and the average parity was 2. Socio-demographic details are displayed in Table 1.

**Table 1:** Results of infertility survey (*N* = 158)

Baseline Characteristics	Frequency (%) or mean ± SD
Age < 35 years	84 (53.5)
Age	35.03 ± (12.24)
University or Post Graduate Education	46 (29)
Have paid employment	92 (59)
Sexuality	
Heterosexual	142 (90)
Homosexual	9 (6)
Bisexual	7 (4)
Pregnant currently	65 (47)
History of difficulty becoming pregnant	16 (11)
History of fertility therapy	4 (3)
Gravidity	3.04 ± 2.39
Parity	2.04 ± 1.62
Number of miscarriage/abortions	1 ± 1.26
Age at first delivery	20.33 ± 5.46

The mean infertility knowledge score was 38.15% for total participants in our study (Tables 2 and 3). Those with a higher level of education (*p* = < 0.0001) and those with paid employment (*p* = 0.01) had a significantly higher level of infertility knowledge (Table 4). The mean positive belief score was 3.31 and the mean negative belief score was 2.96 in our study (Table 2). Those who had a history of infertility treatment had significantly stronger agreement with negative treatment beliefs (*p* = 0.01) (Table 4). There was no significant difference in infertility knowledge or treatment beliefs based on age, sexuality, parity or being pregnant at the time of survey completion. There was no correlation between the fertility knowledge score and having a stronger agreement with negative or positive treatment beliefs. Fertility knowledge and treatment beliefs in relation to socio-demographic factors are displayed in Table 4. The frequency and percentage of participants who correctly answered each item in the knowledge section of the survey is displayed in Table 3.

**Table 2:** Fertility knowledge and treatment beliefs total results

Survey Section	Mean ± SD
Fertility Knowledge	38.15 ± 20.36
Positive beliefs	3.31 ± 0.90
Negative beliefs	2.96 ± 0.88

**Table 3:** Frequency and percentage of participants who correctly answered each knowledge item

Question	Total	Frequency	Percentage (95% CI)
1. A woman is less fertile after the age of 36 years. (True)	157	38	24.2 (17.7–31.7)
2. A couple would be classified as infertile if they did not achieve a pregnancy after 1 year of regular sexual intercourse (without using contraception). (True)	156	59	37.8 (30.2–46.0)
3. Smoking decreases female fertility. (True)	157	55	35.0 (27.6–43.0)
4. Smoking decreases male fertility. (True)	158	39	24.7 (18.2–32.2)
5. About 1 in 10 couples are infertile. (True)	157	89	56.7 (48.6–64.6)
6. If a man produces sperm he is fertile. (False)	157	70	44.6 (36.7–52.7)
7. These days a woman in her 40s has a similar chance of getting pregnant as a woman in her 30s. (False)	157	18	11.5 (6.9–17.5)
8. Having a healthy lifestyle makes you fertile. (False)	157	31	20.0 (13.8–26.8)
9. If a man has had mumps after puberty he is more likely to later have a fertility problem. (True)	157	67	42.7 (34.8–50.8)
10. A woman who never menstruates is still fertile. (False)	158	70	44.3 (36.4–52.4)
11. If a woman is overweight by more than 28 pounds then she may not be able to get pregnant. (True)	158	90	57.0 (48.9–64.8)
12. If a man can achieve an erection then it is an indication that he is fertile. (False)	158	83	52.5 (44.5–60.5)
13. People who have had a sexually transmitted disease are likely to have reduced fertility. (True)	158	72	45.6 (37.6–53.7)

**Table 4:** Two sample T Test/ANOVA to test mean scores for fertility knowledge and treatment belief (*N* = 158)

	Fertility knowledge mean score (SD)	*p*-value	Negative treatment belief mean score (SD)	*p*-value	Positive treatment belief mean score (SD)	*p*-value
Age		0.56		0.05		0.07
< 35 years	37.41 (18.36)		2.81 (0.89)		3.44 (0.77)	
≥35 years	39.32 (22.50)		3.10 (0.82)		3.16 (1.02)	
Education		< 0.0001*		0.08		0.26
University or post graduate	47.55 (23.25)		3.15 (0.88)		3.44 (0.73)	
High school graduate or less	33.98 (17.53)		2.87 (0.88)		3.25 (0.97)	
Paid employment		0.01*		0.9		0.58
Yes	41.58 (20.84)		2.97 (0.97)		3.34 (0.82)	
No	33.06 (18.78)		2.95 (0.75)		3.26 (1.01)	
Have sexual relationship with				0.94		0.05
Males	38.88 (20.83)	0.35	2.95 (0.85)		3.25 (0.89)	
Females	29.06 (13.75)		2.97 (1.37)		3.56 (0.82)	
Both	35.16 (15.92)		3.07 (0.92)		4.07 (0.93)	
Pregnant now		0.76		0.22		0.98
Yes	37.20 (20.25)		2.83 (0.77)		3.25 (0.84)	
No	38.25 (20.88)		3.01 (0.93)		3.25 (0.93)	
Deliver baby before		0.66		0.41		0.79
Yes	38.22 (20.12)		2.93 (0.89)		3.26 (0.91)	
No	36.12 (23.01)		2.81 (0.56)		3.20 (0.80)	
Have difficulty becoming pregnant		0.75		0.84		0.2
Yes	36.54 (22.21)		2.98 (0.90)		3.54 (0.66)	
No	38.30 (20.44)		2.93 (0.86)		3.22 (0.90)	
Require fertility therapy		0.17		< 0.0001*		0.25
Yes	51.92 (26.18)		4 (0.20)		3.75 (1.26)	
No	37.70 (20.37)		2.9 (0.85)		3.24 (0.87)	

*Significant statistical difference was detected

A multiple linear regression analysis was performed to examine the individual effect of different factors by controlling other factors for positive beliefs, negative beliefs and knowledge (Table 5). Based on the multiple linear regression analysis, for negative infertility treatment beliefs, having a history of infertility treatment remained a significant factor. For positive infertility treatment beliefs, age remained a significant factor. Lastly, for infertility knowledge, education remained a significant factor. Results of the multiple linear regression are displayed in Table 5.

**Table 5:** Multiple regression model outcomes for infertility beliefs and knowledge

	Negative belief		Positive belief		Knowledge	
Source	*F* Value	*P* value	*F* Value	*P* value	*F* Value	*P* value
Age	2.74	0.1009	8.82	0.0036*	0.74	0.3908
Education	0.8	0.4956	1.33	0.269	3.85	0.0112*
Work	0.09	0.7655	0.41	0.521	2.94	0.0889
Sexuality	1.92	0.1506	2.64	0.076	0.22	0.8004
Currently Pregnant	0.23	0.6304	0.63	0.4275	0.48	0.4911
Infertility History	2.57	0.1115	0.18	0.6717	1.81	0.1807
Infertility Treatment History	4.78	0.0308*	0.82	0.3675	1.44	0.2319
Parity	0.13	0.7179	0.55	0.4616	0.16	0.6925

The outcome of the multiple regression model was performed on negative beliefs positive beliefs and knowledge separately. The input variables include age, education level, employment, gender, current pregnancy status, Infertility History, Infertility Treatment history and parity. *Significant statistical difference was detected

## Discussion

### Infertility knowledge

Despite being disproportionately affected by infertility, African American women seem to have a limited level of infertility knowledge. In a comparison to the original study by Bunting et al., our participants had a lower level of infertility knowledge (score 38%) when compared to their total number of women (*n* = 8355, average score 59.1%, 79 countries). Our participants also had a lower knowledge score in comparison to their female participants specifically representing the USA (*N* = 427, average score approximately 65%) [9].

While infertility is a disease of many etiologies, a commonly cited cause of infertility among AA women is tubal factor, which is often secondary to a history of sexually transmitted infection or pelvic inflammatory disease [5]. Interestingly, only 45.6% of total participants in our study knew that sexually transmitted diseases could adversely affect fertility. This reflects a concerning lack of sexual health awareness among the women in our study. Our results are consistent with those found by Deatsman et al., who found that AA women were less aware, in comparison to other racial groups, that a history of STI can be risk factor for infertility [10].

As delayed childbearing has become the norm in our society, patient awareness of how age affects infertility is becoming increasingly more important [11]. In our study, 24% percent of participants knew that a woman’s fertility decreases after the age of 36 years. Furthermore, only 11.5%of subjects knew that the probability of conceiving is different between a woman age 30 vs. 40 years. This was also consistent with Deatsman et al’s study, showing AA women were significantly less aware of the impact age has on infertility [10].

Obesity (defined as BMI > 30) is another health condition disproportionately affecting African Americans in the US. In the US approximately 56.5% of African American women are obese compared to 35.3% of Caucasian American women. When including those who are overweight (defined as BMI 25–30), the percentage surges to 82% of AA women and 63.5% of CA women [12]. The survey instrument included the threshold of “greater than 28 pounds overweight”, as this was previously determined in a preliminary study by Bunting et al., to be the point at which there is a significant association with infertility [13]. In our study, 57% of participants correctly associated increased difficulty with fertility and being overweight by > 28 lbs. This is of clear concern as obesity is a modifiable risk factor and the vast majority of AA women have a BMI > 25 [12].

### Infertility treatment beliefs

Overall, our subjects had a relatively neutral response to both negative and positive treatment beliefs. There was no significant difference in treatment beliefs based on age, education level, and parity. Interestingly, those with a history of infertility therapy were significantly more likely to agree with negative treatment beliefs. Detailed questions to characterize the experiences of those who reported a history of infertility treatment were not included in the survey. Therefore, this finding may be due to a personal history of poor success with treatment. However, this finding also raises questions of how their infertility care experiences may differ from other racial groups. It is important to consider the commonly found mistrust of the US healthcare system held among many AAs. This general mistrust is often attributed to the AA response to institutionalized racism and the history of medical maltreatment in the US [14]. It is possible that these negative beliefs result from underlying mistrust rooted in AA history.

### Strengths and limitations

Our study is unique because it includes solely women who self-identify as African-American or black, which is currently the third largest racial ethnic group in the US [15]. This group is often underrepresented in the literature, especially in qualitative studies focused on infertility. Further strengths include the diversity of our subjects, with respect to age, parity and education levels.

There were limitations to this study, which may affect the generalizability of our results. Our survey was administered to a convenience sample at a single location in a safety net hospital. The results of our study may not reflect the infertility knowledge and beliefs of privately insured AA women, those with a higher socioeconomic status or those living in the suburbs, rural or other urban areas. We did not include insurance type as a part of the survey instrument. Therefore, a sampling bias may have been created as the majority of the patients at our clinic are insured by Medicaid or are uninsured. Though we are unable to incorporate specific insurance types per participant, we were able to calculate the proportion of insurance types used across our entire clinic patient population (Fig. 1). Also not included in the survey instrument, were detailed questions to characterize experiences of those who reported a history of infertility treatment.

Unfortunately, the participation rate was not tracked and therefore cannot be calculated or analyzed. However, most patients who were invited to participate in this study completed the survey. Additionally, a sample selection bias may have been created on exceptionally busy clinic days when recruitment occurred less often. Lastly, the sociodemographic composition of our patient population differed greatly from the original study and the proportion of African American representation in the original study was not published. Due to this, we were unable to make a stronger/statistical comparison. Though this is a limitation, it also underscores our disposition to further characterize this subpopulation’s perspective of infertility.

## Conclusion

Our study contributes a unique perspective of a significant yet underrepresented population of American women. Numerous studies have confirmed that there is a racial disparity for infertility, which prevails through access, pursuit, utilization and success rates of treatment [4–7]. Our study shows that African American women within our healthcare setting have a limited level of infertility knowledge. The consistent finding of poor infertility knowledge is of particular importance because being able to understand the basics of fertility and reproduction can shape a woman’s fertility decision-making and planned childbearing.

One of the biggest modifiable factors for the disparities associated with infertility is likely knowledge. Regardless of socioeconomic status, women’s health providers should routinely include culturally sensitive fertility awareness and education for all of their patients. Both the American College of Obstetricians and Gynecologists and the Centers for Disease Control and Prevention recommend inclusion of “Reproductive Life Planning” as part of the annual well woman visit [16]. Additional educational outreach modalities include infertility workshops, brochures, online modules and smartphone mobile applications. A worthwhile future investigation would be to incorporate a wider sociodemographic patient population, provide the aforementioned educational modalities and compare pre and post-test knowledge and beliefs.

Though increased awareness and education is likely a promising preventative strategy, the disparity in infertility care should not be ignored. The full spectrum of infertility care is not commonly provided in low-income communities. These services are also not readily available at our hospital. Hence, clear patient inequities are easily appreciated in the attempt to counsel those who require ART for their infertility but are unable to afford subspecialty fertility care elsewhere. One strategy to correct this issue would be through the implementation of more low-cost IVF programs. With the creation of low-cost yet high quality ovarian stimulation protocols and IVF techniques, the prospect of providing full spectrum infertility care to those living in low resource settings has become feasible [17]. As the outcomes of these new techniques and programs materialize in the literature, hopefully additional programs with similar goals will begin to disseminate throughout the US and worldwide.

In conclusion, African American women in our urban clinic setting seem to have a limited level of knowledge pertaining to infertility. This finding is likely generalizable to other minority groups within similar settings and warrants a great need for further research, as well as effective, culturally relevant educational interventions. It is our responsibility, as women’s health providers, to work in unison not only to improve patient awareness but to also strive for changes that will ensure equal quality infertility care for all.

## Additional file

Additional file 1: Infertility Survey. (DOCX 17 kb)

## Data Availability

The datasets during and/or analyzed during the current study are available from the corresponding author on reasonable request.
