# Supplementary material for: Infertility knowledge and treatment beliefs among African American women in an urban community
Source: Contracept Reprod Med. 2019 Sep 24;4:16. doi: 10.1186/s40834-019-0097-x (PMC6757383; doi:10.1186/s40834-019-0097-x)
Supplement: Supplementary file 1 — Infertility Survey. (DOCX 17 kb) [file 40834_2019_97_MOESM1_ESM.docx]

**Infertility Survey**

***Demographics:***

1. Age_________
2. Ethnicity/Heritage: (circle all that apply)
   1. Black
   2. White
   3. Hispanic
   4. Asian
   5. Other: __________
3. Highest education Level
   1. Less than high school diploma
   2. High school diploma
   3. University
   4. Post-graduate
4. Do you have paid employment? Yes/No
5. Who do you have sexual relations with?
   1. Males
   2. Females
   3. Both

5. Obstetrical History

- 1. How many times have you been pregnant: _____
  2. How many deliveries have you had: ______
  3. How many miscarriages/abortions have you had: ____
  4. Are you currently pregnant? Yes_____ No______
  5. How old were you when you had your children? _____,____,____,____,____
  6. Did you have difficulty becoming pregnant? Yes_____ No_____
  7. Did you require fertility therapy? Yes____ No______

***Knowledge***

Instructions: below are some statements concerning fertility. Please indicate whether you believe the statements are TRUE or FALSE of fertility by circling the appropriate answer. If you do not know the answer please circle DON’T KNOW.

1. A woman is less fertile after the age of 36 years.

TRUE/FALSE/DON’T KNOW

1. A couple would be classified as infertile if they did not achieve a pregnancy after 1 year of regular sexual intercourse (without using contraception).

TRUE/FALSE/DON’T KNOW

1. Smoking decreases female fertility.

TRUE/FALSE/DON’T KNOW

1. Smoking decreases male fertility.

TRUE/FALSE/DON’T KNOW

1. About 1 in 10 couples are infertile.

TRUE/FALSE/DON’TKNOW

1. If a man produces sperm he is fertile.

TRUE/FALSE/DON’T KNOW

1. These days a woman in her 40s has a similar chance of getting pregnant as a woman in her 30s.

TRUE/FALSE/DON’T KNOW

1. Having a healthy lifestyle makes you fertile.

TRUE/FALSE/DON’T KNOW

1. If a man has had mumps after puberty he is more likely to later have a fertility problem.

TRUE/FALSE/DON’T KNOW

1. A woman who never menstruates is still fertile.

TRUE/FALSE/DON’T KNOW

1. If a woman is overweight by more than 28 pounds then she may not be able to get pregnant.

TRUE/FALSE/DON’T KNOW

1. If a man can achieve an erection then it is an indication that he is fertile. TRUE/FALSE/DON’T KNOW
2. People who have had a sexually transmitted disease are likely to have reduced fertility.

TRUE/FALSE/DON’T KNOW

***Treatment Beliefs***

Instructions: Please circle on a scale of 1-5 based on how you feel about the following statements. 1= strongly disagree to 5= strongly agree

1. Fertility treatment is very safe

1 2 3 4 5

1. most people who start fertility treatment eventually get pregnant

1 2 3 4 5

1. Fertility treatment is a scary experience

1 2 3 4 5

1. Fertility treatment may have short-term physical effects (e.g. headaches, nausea)

1 2 3 4 5

1. Long-term term physical effects (e.g. cancer)

1 2 3 4 5

1. Fertility treatment can cause emotional problems.

1 2 3 4 5
